# Tunnelling of electrons via the neighboring atom

**DOI:** 10.1038/s41377-023-01373-2

**Published:** 2024-01-16

**Authors:** Ming Zhu, Jihong Tong, Xiwang Liu, Weifeng Yang, Xiaochun Gong, Wenyu Jiang, Peifen Lu, Hui Li, Xiaohong Song, Jian Wu

**Affiliations:** 1 School of Physics and Optoelectronic Engineering, Hainan University, Haikou, 570288 China; 2 School of Information and Communication Engineering, Hainan University, Haikou, 570288 China; 3 State Key Laboratory of Precision Spectroscopy, East China Normal University, Shanghai, 200241 China; 4 Center for Theoretical Physics, Hainan University, Haikou, 570288 China

**Keywords:** Ultrafast photonics, Nonlinear optics

## Abstract

As compared to the intuitive process that the electron emits straight to the continuum from its parent ion, there is an alternative route that the electron may transfer to and be trapped by a neighboring ionic core before the eventual release. Here, we demonstrate that electron tunnelling via the neighboring atomic core is a pronounced process in light-induced tunnelling ionization of molecules by absorbing multiple near-infrared photons. We devised a site-resolved tunnelling experiment using an Ar-Kr^+^ ion as a prototype system to track the electron tunnelling dynamics from the Ar atom towards the neighboring Kr^+^ by monitoring its transverse momentum distribution, which is temporally captured into the resonant excited states of the Ar-Kr^+^ before its eventual releasing. The influence of the Coulomb potential of neighboring ionic cores promises new insights into the understanding and controlling of tunnelling dynamics in complex molecules or environment.

## Abstract (web summary)

A van der Waals complex with an internuclear distance of 0.39 nm is designed as a prototype system to track electron tunneling via neighboring atoms within a sub-nanometer scale.

## Graphical abstract

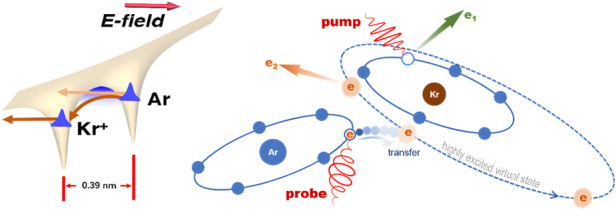

## Introduction

Tunnelling is one of most fundamental processes in quantum mechanics, where the wave packet could traverse a classically insurmountable energy barrier with a certain probability. Within atomic scale, tunnelling plays a significant role in molecular biology, such as speeding up an enzymatic catalysis^1–3^, promoting spontaneous mutations in DNA^4–7^, and triggering a signaling cascade of olfactory^8^. Moreover, for devices such as optoelectronic chips, whose size has been already close to the sub-nanometer atomic scale, the tunneling effect is also significant. Therefore, exploring the real-time imaging of electron tunneling dynamics in complex structures is of great importance not only for the fundamental physics, but also for the development of tunnel transistors and ultrafast optoelectronic devices. On the other hand, the optical field-induced electron motion is the key process of light-induced chemical reaction^9^, charge and energy transfer^10^, and photoelectron tunnelling^11–17^ and radiation emission^18–20^. In a complex environment^21,22^, the potential effects of neighboring ionic cores have significant influence on the motion of electrons, such as the intramolecular charge rearrangement^23,24^, the internuclear electron charge transfer^25–27^ in complex clusters. So far, the role of neighboring atomic cores to electron tunnelling dynamics is still open.

In this work, we designed a van der Waals complex Ar-Kr^+^ as a prototype system with an internuclear distance of 0.39 nm to track the electron tunneling via the neighboring atom in the system of sub-nanometer scale. The intrinsic electron localization of the highest occupied molecular orbital of Ar-Kr^28,29^ gives a preference of electron removal from Kr site in the first ionization step. The site assisted electron hole in Ar-Kr^+^ guarantees that the second electron is mainly removed from the Ar atom in the second ionization step, where the electron may straightly tunnel to continuum from the Ar atom or alternatively via the neighboring Kr^+^ ionic core. The molecular orientation can be identified by measuring the ejection direction of the nuclear fragments under the recoil axial approximation. Therefore, by measuring the nuclear fragments of Ar^+^ and Kr^+^ and two electrons ejected from a doubly ionized Ar-Kr dimer in coincidence, we can retrieve the tunnelling site and the releasing order of two electrons in the molecular frame, which allows us to interrogate the role of the neighboring ionic Coulomb potential in electron tunnelling. These results on how electrons tunnelling between atoms in such extremely small complex will provide us with a valuable experimental platform for studying the fundamental principles of quantum mechanics.

## Results

**Fig. 1:**
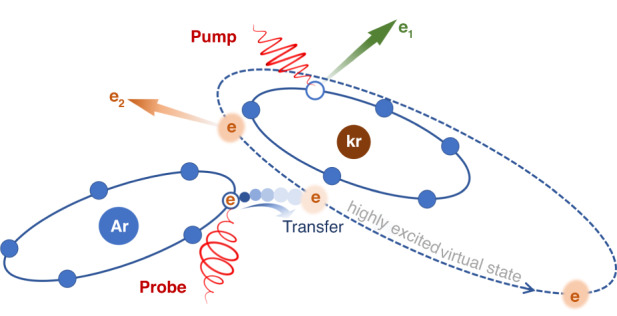
Tunnelling of electron via the neighboring atom in strong-field ionization of a dimer. The electron emitted from Ar atom is firstly trapped to the highly excited transient states of the Ar-Kr^+*^ before its eventual releasing to the continuum. A linearly polarized pump laser pulse is used to prepare the Ar-Kr^+^ ion by removing *e*_1_ from Kr site, and a time-delayed elliptically polarized probe laser pulse is used to track the electron transfer mediated electron tunnelling dynamics (*e*_2,_ orange arrow)

We focus on the intriguing electron transfer mediated tunnelling dynamics in Ar-Kr^+^ as illustrated in Fig. 1. A substantial probability of electron wave packet from Ar induced by the Coulomb attraction of Kr^+^ tunnels through the classical-forbidden barrier between two ionic cores. Subsequently, the electron wave packet is resonantly captured by the system before it is released to the continuum. The neighboring Kr^+^ acts as a resonance reservoir for this internuclear electron transfer-mediated tunnelling process. We experimentally probe this effect by tagging the emission direction of the photoelectron in the polarization plane of the elliptically polarized near-IR femtosecond laser pulse and observing the reduction of its transverse momentum distribution along the light propagation direction. An improved-Coulomb-corrected strong-field approximation (ICCSFA) theoretical method is developed to numerically simulate the intriguing electron tunnelling dynamics^30^. We find that the electron tunnelling via the neighboring atom is a general process in strong-field ionization of molecules by absorbing multiple near-infrared photons.

**Fig. 2:**
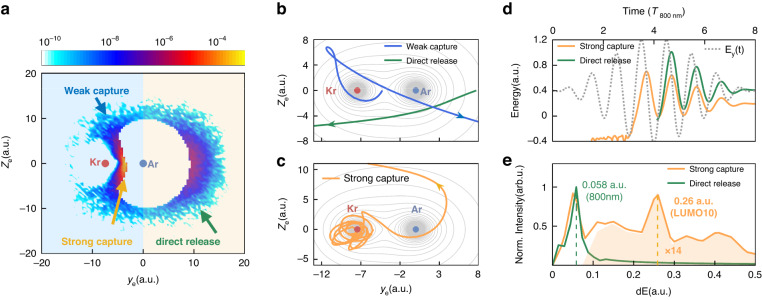
Simulation electron trajectories. **a** The ICCSFA calculated initial tunneling exit distribution of the electron emitted from the Ar. The yellow and blue areas correspond to the direct release and tunnelling capture regions. The nuclear position of the Ar and Kr are labelled by the colored dots. **b**, **c** The typical direct release (green line), weak capture (blue line) and strong capture (orange line) trajectories of the electrons born in **a**. The gray contour lines indicate the Coulomb potential well of the Ar^+^-Kr^+^. **d** Time-dependent streaking energy spectra of the electrons born in the strong capture and direct release region. The dash gray line plots the electric field projection along the major axis of the elliptically polarized probe pulse. **e** The FFT amplitude distribution of the time-dependent streaking energy spectra in **d**. The amplitude of the fast wiggle is enlarged 14 times to increase the visibility

Figure 2a shows the tunnelling exit distribution of the electron from the strong-field ionization of Ar-Kr^+^ ion simulated by the ICCSFA method. The nuclear axis of Ar-Kr^+^ is orientated with the Ar and Kr^+^ at the (0.0, 0.0) and (−7.45, 0.0) of the coordinate system of (*y*_e_, *z*_e_), where *y*_e_ and *z*_e_ are the coordinate axes along the major and minor axes of the elliptically polarized laser pulse. The false color scale stands for the ionization rate. A bright tunnelling burst is observed between the Ar^+^ and Kr^+^ ionic cores, in contrast to the electron directly tunnels to the continuum from the Ar site. The neighboring ionic Coulomb potential of the Kr^+^ alters not only the propagation of outgoing electron in continuum, but also the enigmatic initial tunnelling dynamics, which cannot be well described in the traditional saddle point equation^31–34^. Our ICCSFA model adapts the ionic Coulomb potential effect to both the tunnelling and the subsequent continuum propagation of the electron (see Supplementary Information, for electron tunnelling exit distribution simulated by the traditional Coulomb-corrected strong-field approximation (CCSFA)).

As depicted in Fig. 2a, we distinguish the electron tunnelling into three regions, i.e., the direct release to the continuum from the Ar site (yellow area), and the strong capture and weak capture regions (blue area) for electron tunnels towards the Kr^+^. We performed an intensity scan with the ICCSFA simulations. The initial tunneling exit distributions of the strong/weak captured and direct released electrons are present for the cases with different laser intensities, which demonstrates the general property of the electron capture (see details in section 2 of the Supplementary Information). To access the details of the tunnelling dynamics, we track the electron trajectory for each individual ionization event. The electron could be emitted at any time within the pulse duration of laser field. Here we choose the typical electron trajectory with a large weight. As shown in Fig. 2b, the green curve shows a typical electron trajectory of the directly released electron. However, for the capture regions, the electron initially tunnelling from the Ar towards the neighboring Kr^+^ is trapped into the highly-excited orbits of the Ar-Kr^+*^, which rotates around the Kr^+^ for many rounds before eventual releasing to the continuum (strong capture region, Fig. 2c), or quickly scattered by the Kr^+^ (weak capture region, Fig. 2b). The strong and weak capture trajectories are mostly released from the ionization bursts between Ar and Kr^+^. As displayed in Fig. 2d, the strong capture process shows a fast wiggle structure with negative total energy due to the trapping of the Kr^+^. Figure 2e shows the evaluated frequency spectrum of the electron trajectories by performing a fast Fourier transformation (FFT) of the time-dependent electron streaking energy spectra of Fig. 2d. The wiggle frequency of dE ~0.058 a.u. corresponds to the electron quiver motion in continuum following the carrier frequency of the driven laser field, whereas the fast wiggle frequency of dE ~ 0.26 a.u. is close to the binding energy of the excited virtual states of the LUMO-9/10 (0.22 ~ 0.27 a.u.) of the Ar-Kr^+^ at the internuclear distance of 7.45 a.u.. The agreement between the frequency of classical trajectories and the binding energy of the virtual states of Ar-Kr^+*^ provides a classical presentation of the quantum resonance ionization scenario^35,36^ which appears above the continuum threshold.

**Fig. 3:**
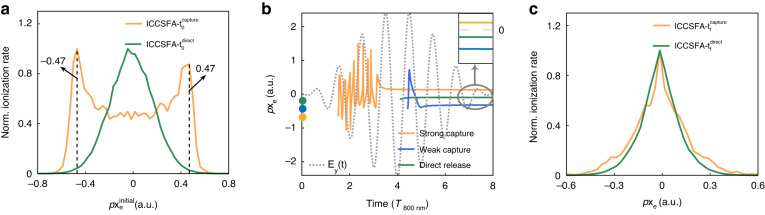
Evolution of transverse momentum. **a** The ICCSFA calculated initial *p*x_e_ distribution of the electron born in the direct (green curve) and capture (orange curve) region. **b** The time-dependent evolution of *p*x_e_ electrons born in the direct release and strong/weak capture regions as shown in Fig. 2b and c. The colored dots at time zero show the *p*x_e_^initial^ of these electrons, and their final *p*x_e_ momenta are highlighted in the inset. **c** The final transverse momentum distribution *p*x_e_ of the electrons born in the direct release region and tunnelling capture region

The trapping effect from the neighboring ionic core can be identified by observing the narrowing of the photoelectron transverse momentum distribution *p*x_e_ along the laser propagation axis, where the streaking effect of the laser field on the electron motion can be avoided. Figure 3a shows the initial transverse momentum distributions, *p*x_e_^initial^, of the electron born in the capture (orange curve) and direct release (green curve) regions. The *p*x_e_^initial^ of the capture region shows a non-zero value with two satellite peaks around |*p*x_e_^initial^ | ~ 0.47 a.u. with respect to the zero-centered distribution of direct release region. As described in Eq. (4) (see Materials and methods), the Coulomb attraction of the neighboring Kr^+^ counteracts the Coulomb potential of Ar^+^. It will induce an additional energy to the tunneled electron, $\Delta {\rm{V}}\left({\rm{r}}\right)=\left|-\frac{{Z}_{{Kr}+}}{\left\langle {r}_{{capt}}\right\rangle -{r}_{{Kr}}}-\frac{{Z}_{{Kr}+}}{{r}_{{Ar}}-{r}_{{Kr}}}\right|$ ~ 0.104 a.u., resulting in the non-zero |*p*x_e_^initial^ | ~ 0.46 a.u.in Fig. 3a, where the two potential terms denote the energy at the tunnelling exit and under tunnelling barrier, $\left\langle {r}_{{capt}}\right\rangle$ is the expectation value of the tunnelling exit of the electrons released from the capture region, ${r}_{{Kr}}$ and ${r}_{{Ar}}$ are the coordinates of Kr^+^ and Ar^+^, respectively. Nevertheless, the electron born in the direct release region only suffers the Coulomb attraction from the Ar^+^, which is identical to the case of electron tunnelling from an atom, leading to the zero concentrated initial transverse momentum distribution. The subsequent propagation of the tunneled electron in the vicinity of the ionic Coulomb potential will narrow the initial transverse momentum distribution^37–40^.

Figure 3b shows the time evolution of *p*x_e_ in the elliptically polarized femtosecond pulse for electrons born in different regions. For the strong capture region, the rapid oscillation of *p*x_e_ at the ascending edge of the laser pulse corresponds to the fast rotation of the trapped electron around Kr^+^ as shown in Fig. 2c, which dramatically increases the interaction time of the electron with the ionic core before its eventual releasing to the continuum. As a result, the electron born in the strong capture region with an initial momentum of *p*x_e_^0^ ~ 0.66 a.u. ends with a final transverse momentum of *p*x_e_^f^ ~ 0.13 a.u., as shown in Fig. 3b. This strong electron capture effect blocked the transferring of its initial momentum at the tunnelling exit to the continuum. It leads to the observed narrowing of the photoelectron momentum distribution along the light propagation direction, as shown in Fig. 3c. The double peaked initial momentum distribution of the tunneled electron in the capture region is shrunk to a distribution concentrated around zero.

**Fig. 4:**
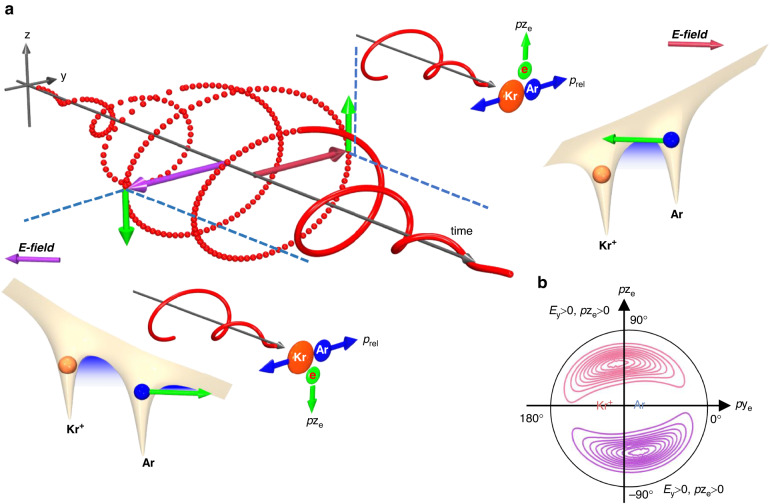
Schematic diagram of the electron tunnelling via neigboring atom. **a** The concept of the angular streaking protocol driven by a clockwise rotating elliptically polarized near-IR femtosecond laser pulse. The yellow surfaces show the laser-field dressed Coulomb potential well of Ar^+^-Kr^+^ after the electron removal from Ar, where the gradually varied blue area between two nuclei indicates the tunnelling exit. The other insets show the final momentum vector correlation between molecular orientation, electron momentum and the instantaneous laser vector. **b** The tunnelling site resolved final photoelectron momentum distribution in the polarization plane, where the red contour curves with *p*z_e_ > 0 corresponds to the electrons freed by laser field pointing from Kr^+^ to Ar, and vice versa

Based on the angular streaking protocol^14,16,41^, the final photoelectron emission direction in the polarization plane is strongly sensitive to the initial electron tunnelling dynamics^42–44^. Here are the different weights and tunnelling exit distributions of direct release electron and strong/weak capture electron depending on their ionization times. Thereby, by gating the eventual emission direction of the electron, we can track back the releasing times of the electron originated from distinct ionization regions, which have different tunnelling trajectories and experience different electron-Coulomb interactions with the ionic cores. As depicted in Fig. 4a, if the electron is released when the instantaneous laser field points from Kr^+^ to Ar (*E*_y_ > 0, laser field clockwise rotating from *E*_z_ to *E*_y_), it will gain a final momentum of *p*z_e_ > 0, in which the electron suffers a strong Coulomb interaction from the neighboring Kr^+^ and belongs to the tunnelling capture region. On the contrary, when the laser field points from Ar to Kr^+^ (*E*_y_ < 0), the electrons are mostly born from direct release region with a final momentum of *p*z_e_ < 0. Figure 4b shows the classically predicted momentum distribution of the released electron. The distribution of *p*x_e_ is thus analyzed for each tagged photoelectron emission direction in the polarization plane, which is defined as *ϕ*_e_ = tan^−1^(*p*z_e_/*p*y_e_), to track back the tunnelling dynamics of the electron born in different regions.

## Discussion

**Fig. 5:**
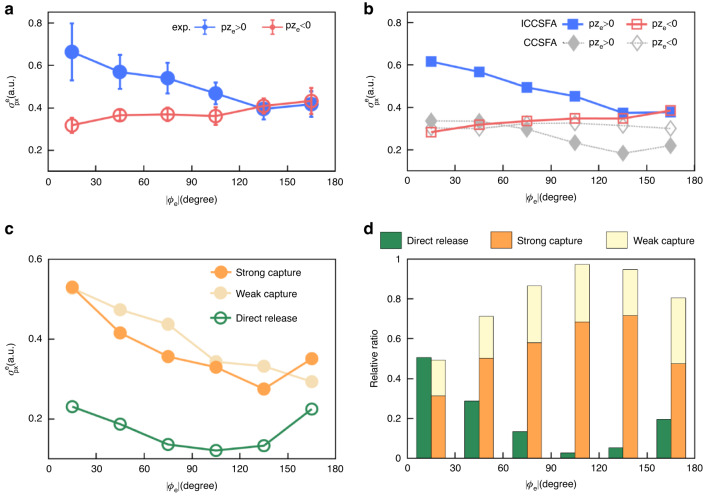
Transverse momentum width distribution. **a**, **b** The measured (**a**) and calculated (**b**) transverse momentum width distribution (${\sigma }_{{px}}^{e}$) as a function of photoelectron emission angle in the polarization plane. The capture dominated *p*z_e_ > 0 range is labelled by solid blue dots, while the *p*z_e_ < 0 range is labelled by open red dots. The gray (open) diamonds show the ${\sigma }_{{px}}^{e}$ distribution calculated by the CCSFA method without including the tunnelling capture effect. **c** The trajectory resolved ${\sigma }_{{px}}^{e}$ distribution as a function of photoelectron emission angle in the capture-dominated *p*z_e_ > 0 range. The strong, weak capture and direct release process are shown in solid orange, light orange circles and open green circles, respectively. **d** The yield ratio of direct release, weak and strong capture trajectories as a function of photoelectron emission angle

By numerically fitting the distribution of *p*x_e_ with the Gaussian function, we obtain the full width at half maximum (FWHM) of the distribution to investigate the narrowing of the transverse momenta. Figure 5a and b show the measured and simulated FWHM, denoted as ${\sigma }_{{px}}^{e}$, of the final distribution of *p*x_e_ as a function of *ϕ*_e_ in the polarization plane with *p*z_e_ > 0 and *p*z_e_ < 0, respectively. As the *ϕ*_e_ increases (or decreases) from 0° to 180° (or -180°), in which each emission angle is integrated over a *ϕ*_e_ spanning window of 30°, the ${\sigma }_{{px}}^{e}$ value decreases (or increases) gradually for the capture-dominated region (solid blue circles) as compared to the direct release region (open red circles). The ion momentum distribution and the integrated angular distribution of the Ar-Kr^+^ dimers are experimentally measured. We choose the |ϕ_ion_^rel^ | ≤30*°*, where ϕ_ion_^rel^ = arctan(px_ion_^rel^/py_ion_^rel^), and p_ion_^rel^ = p_ion_^Ar+^-p_ion_^Kr+^. The molecular orientation angle range in theoretical simulations was performed by integrating overall orientation angles according to the ionization rate of each molecular orientation. As shown in Fig. 5, the ICCSFA calculated pattern of the angular resolved transverse momentum distribution agrees very well with the experimental measurement. As shown in Fig. 5b (gray diamonds), the ${\sigma }_{{px}}^{e}$ from the traditional CCSFA without including the crucial role of the neighboring atomic core noticeably deviates from the experimental results, confirming the important role of electron transfer mediated tunnelling dynamics in our experiment. Since the width of the final *p*x_e_ is tightly related to the relative ratio between electrons born in the direct release, strong and weak capture regions, we can disentangle the final ${\sigma }_{{px}}^{e}$ formulated as ${\sigma }_{x}=(\sum _{i}{\sigma }_{{xi}}{Y}_{i}^{{direct}}+\sum _{j}{\sigma }_{{xj}}{Y}_{j}^{{strong}}+\sum _{k}{\sigma }_{{xk}}{Y}_{k}^{{weak}})/(\sum _{i}{Y}_{i}^{{direct}}+\sum _{j}{Y}_{j}^{{strong}}+\sum _{k}{Y}_{k}^{{weak}})$, where ${Y}_{{xi},j,k}$ and ${\sigma }_{{xi},j,k}$ is the ionization rate and the width of the *p*x_e_ of the direct release, strong and weak capture electrons, respectively. Figures 5c and d show the *p*x_e_ width and relative ratios of different pathways as a function of *ϕ*_e_ in the capture dominated range of *p*z_e_ > 0. As depicted in Fig. 5c, the widths of the *p*x_e_ of the direct release electrons (mainly given by the initial transverse momenta at the tunnelling exit, green circles) are much narrower than those of the strong and weak capture electrons (orange circles). Since the CCSFA cannot properly describe the electron capture process, the resulted ${\sigma }_{{px}}^{e}$ (Fig. 5b, gray diamonds) are always smaller than those of the ICCSFA simulation (Fig. 5b, blue squares) and experimental observation (Fig. 5a, blue circles). By including the electron capture process induced by the neighboring ionic core, the ${\sigma }_{{px}}^{e}$ of the ICCSFA simulation agrees very well with the experimental results for different emission directions (Fig. 5a and b). As summarized in Fig. 5d, the ICCSFA simulated results further count a probability ~54% of the electrons born in the strong capture region and tunneled via the neighboring Kr^+^ under the nuclear orientation as presented in Fig. 2a.

To conclude, we experimentally and theoretically investigate an electron transfer-mediated tunnelling dynamics in a site-resolved Ar-Kr^+^ prototype system. We demonstrate that owning to the Coulomb interaction of the neighboring atom, the electron wave packet can penetrate through the barrier between the two cores with a noticeable probability and can be trapped in the intermediate excited states with few femtoseconds or couple attoseconds before eventually released to the continuum. The utilization of few-cycle pulses and time-gate technique is anticipated to enable a discerning distinction between strong and weak capture electrons. The ICCSFA theory well describes the electron tunnelling dynamics in this heteronuclear diatomic system by carefully modeling the role of neighboring Coulomb potential in the saddle point equation. Our findings provide a new insight into the key role of neighboring Coulomb potential in the under barrier electron tunnelling dynamics^45–47^, high harmonic generation in solid state^48^, and provide a new route to probe and control the tunnelling dynamics in complex biological molecules.

## Materials and methods

### Experimental setup

A mixture of rare gases of He, Ar and Kr with a ratio of 1:7:2 and a driving pressure of 3-bar was used to generate the ArKr dimer via the supersonic expansion through a 30-μm nozzle. The ultrashort femtosecond laser pulse (25 fs, 790 nm, 10 kHz) was split into two pulses by using a noncolinear Mach-Zehnder interferometer. The pump and probe laser pulses were tightly focused onto the supersonic gas jet by using a concave sliver mirror (*f* = 7.5 cm) inside the reaction microscope of cold target recoil ion momentum spectroscopy (COLTRIMS)^49–51^. The polarization states, relative time delay and intensities of the pump and probe pulse were finely tuned via a half- and a quarter-waveplate, a delay stage and a neutral filter. The peak intensities of the pump and probe laser pulse and the ellipticity of the probe pulse in the interaction region were estimated to be *I*_pump_ ~ 0.75 × 10^14 ^W/cm^2^, and *I*_probe_ ~ 0.85 × 10^14 ^W/cm^2^, and *ε*_probe_ = 0.8, respectively. The pump pulse is linearly polarized along *y*, and the elliptically polarized probe pulse has its major and minor polarization axes along *y* and *z*. The laser field of the probe laser pulse rotated clockwise from +*z* to +*y* in the *y*-*z* (polarization) plane and propagated along -*x* axis after the focusing mirror. The photoionization-created photoelectron and nuclear fragments were accelerated and guided by a homogeneous electric field and magnetic field towards the time- and position-sensitive detectors at the opposite site of the spectrometer.

The linearly polarized pump laser pulse removes one electron from Kr site of the Ar-Kr dimer creating a concentrate electron momentum distribution along z axis, |*p*z_e_ | < 0.3 a.u., associated with the generation of Ar-Kr^+^ ion. Subsequently, the elliptically polarized probe pulse releases the second electron from the Ar site of the dimer and deflects the final photoelectron momentum in the *y*-*z* plane with a larger *p*z_e_ momenta than those emitted by the pump laser pulse owning to the angular streaking. The sequential double ionization of the ArKr gives rise to the Coulomb exploding channel Ar^+^ + Kr^+^ + *e*_1_ + *e*_2_. The internuclear distance between Ar and Kr, ~ 7.45 a.u., is given by the bond length at the time of the second electron removal. Meanwhile, the pump and probe laser pulses are delayed by 300-fs to avoid the rotational revival period of the ArKr nuclear wave packet. The single pump or probe laser pulse is not intense enough to induce an efficient double ionization of ArKr, which manifests that the gated double ionization channel was generated via the sequential interaction of the dimer with the pump and probe laser pulses.

### Theoretical method

In strong-field approximation (SFA), the transition matrix element in the length gauge is given by

1 $${M}_{{\bf{p}}}\left({t}_{f},{t}_{i}\right)=-i{\int }_{{t}_{i}}^{{t}_{f}}d\tau \left\langle {\boldsymbol{p}}+{\boldsymbol{A}}(\tau ){\rm{|}}{\boldsymbol{r}}\cdot {\boldsymbol{E}}(\tau ){\rm{|}}{\psi }_{0}\right\rangle {e}^{i{S}_{{I}_{p},{\bf{p}}}(\tau )}$$

Since the action ${S}_{{I}_{p},{\bf{p}}}(\tau )$ is a rapidly oscillating function of *τ*, we can apply the asymptotic expansion to approximately calculate the complex integral under the saddle-point approximation. The time integral in the SFA matrix element is integrated over all saddle-points {*t*_s_^(α)^},

2 $${M}_{{\bf{p}}}=-\frac{\kappa }{\sqrt{2}}\mathop{\sum }\nolimits_{\alpha }\frac{{e}^{i{S}_{{I}_{p},{\bf{p}}}\left({t}_{s}^{\left(\alpha \right)}\right)}}{{S}_{{I}_{p},{\bf{p}}}^{{\prime\prime} }({t}_{s}^{(\alpha )})}$$

where $\kappa =\sqrt{2{I}_{p}}$ denotes the characteristic momentum of a bound electron, ${S}_{{I}_{p},{\bf{p}}}^{{\text{'}\text{'}}}({t}_{s}^{\left(\alpha \right)})$ denotes the second-order time derivative of the action ${S}_{{I}_{p},{\bf{p}}}({t}_{s}^{\left(\alpha \right)})$. Meanwhile, the *α*^th^ saddle-point *t*_s_^(α)^ satisfies the saddle-point equation (SPE),

3 $${\left.\frac{\partial {S}_{{I}_{p,{\bf{p}}}}}{\partial t}\right|}_{{t}_{s}^{\left(\alpha \right)}}=0\Rightarrow {\frac{1}{2}\left({\bf{p}}+{\bf{A}}\left({t}_{s}^{\left(\alpha \right)}\right)\right)}^{2}=-{I}_{p}$$

where ***A***(*t*) is the vector potential of the laser field, *t*_*s*_ *= t*_*r*_ + *i·t*_*i*_ is the complex time of the saddle point. In traditional semiclassical strong-field ionization methods, including the fundamental strong-field approximation and Coulomb-corrected strong-field approximation (CCSFA), the electron tunnelling dynamics under barrier is defined by the SPE. The ionization potential *I*_*p*_ is defined as the total energy difference between the final ionic state and the initial ground state under the energy conservation law. This definition takes a mandatory assumption that the electrons were fully released to continuum state without any influence from the ionic potential well after the ionization transition and without any further Coulomb effect from neighboring ionic cores.

To accurately model the electron tunnelling dynamics via neighboring Coulomb potential, we develop a model of Improved-Coulomb-corrected strong-field approximation (ICCSFA) by adapting the Coulomb potential effect in the SPE,

4 $$\frac{1}{2}{({\boldsymbol{p}}+{\boldsymbol{A}}({t}_{s}))}^{2}+V\left[{\boldsymbol{r}}\left({t}_{s}\right)\right]=-{I}_{p}-V\left[{\boldsymbol{r}}\left({t}_{r}\right)\right]$$

where *t*_*s*_ and *t*_*r*_ present the imaginary time of the tunnelling and real-time in the tunnelling exit, $V\left[{\boldsymbol{r}}\left({t}_{s}\right)\right]=-\frac{{Z}_{{Ar}}}{{\rm{|}}{\boldsymbol{r}}\left({t}_{s}\right)-{r}_{{Ar}}{\rm{|}}}-\frac{{Z}_{{Kr}}}{{\rm{|}}{\boldsymbol{r}}\left({t}_{s}\right)-{r}_{{Kr}}{\rm{|}}}$ and $V\left[{\boldsymbol{r}}\left({t}_{r}\right)\right]=-\frac{{Z}_{{Ar}}}{{\rm{|}}{\boldsymbol{r}}\left({t}_{r}\right)-{r}_{{Ar}}{\rm{|}}}-\frac{{Z}_{{Kr}}}{{\rm{|}}{\boldsymbol{r}}\left({t}_{r}\right)-{r}_{{Kr}}{\rm{|}}}$ define the Coulomb correction under the tunnel barrier and at the tunnelling exit, respectively. The adapted SPE (4) describes the energy conservation during the ionization transition process when the bound electron tunnels through the Coulomb potential barrier of the diatomic molecules deformed by the intensely external optical field. Based on the ICCSFA, all the initial velocity, tunnelling exit, ionization time of the electrons emerging at the continuum and the weight of the electron trajectories are accessible.

Furthermore, the motion of the electron in the continuum is determined by Newton’s equations,

5 $$\left\{\begin{array}{c}\dot{{\bf{r}}}\left(t\right)={\bf{p}}\left(t\right)\\ \dot{{\bf{p}}}=-\frac{{Z}_{Ar}\,\cdot\, \left({\bf{r}}\left(t\right)-{{\bf{r}}}_{{Ar}}\right)}{{\left|{\bf{r}}\left(t\right)-{{\bf{r}}}_{{Ar}}\right|}^{3}}-\frac{{Z}_{{Kr}}\,\cdot\, \left({\bf{r}}\left(t\right)-{{\bf{r}}}_{{Kr}}\right)}{{\left|{\bf{r}}\left(t\right)-{{\bf{r}}}_{{Kr}}\right|}^{3}}-{\bf{E}}\left(t\right)\end{array}\right.$$

The ordinary differential Eq. (5) can be solved by using a fourth-order Runge-Kutta method. The classical action, i.e., the phase of each electron trajectory is as following,

6 $${S}_{{{I}_{p},{\bf{v}}}_{{T}_{p}}}\left({t}_{r}^{\left(\alpha \right)},{T}_{p}\right)={\int }_{\!{t}_{r}^{\left(\alpha \right)}}^{{T}_{p}}\left(\frac{1}{2}{{\bf{v}}}^{2}\left(\tau \right)-\frac{{Z}_{{Ar}}}{\left|{\bf{r}}\left(t\right)-{{\bf{r}}}_{{Ar}}\right|}-\frac{{Z}_{{Kr}}}{\left|{\bf{r}}\left(t\right)-{{\bf{r}}}_{{Kr}}\right|}+{I}_{p}\right)d{\tau }$$

where **v**(*τ*) is the instantaneous velocity of the electron, and *T*_p_ is the switched-off time of laser field.

### Supplementary information

Supplementary Information for Tunnelling of electrons via the neighboring atom
