# Supplementary material for: Tunnelling of electrons via the neighboring atom
Source: Light Sci Appl. 2024 Jan 16;13:18. doi: 10.1038/s41377-023-01373-2 (PMC10791752; doi:10.1038/s41377-023-01373-2)
Supplement: Supplementary file 1 — Supplementary Information for Tunnelling of electrons via the neighboring atom [file 41377_2023_1373_MOESM1_ESM.docx]

Supplementary Information for

**Tunnelling of electrons via the neighboring atom**

**Ming Zhu,^†,‡,§^ Jihong Tong,^¶,§^ Xiwang Liu,^†^ Weifeng Yang,^†,*,**^ Xiaochun Gong,^¶^ Wenyu Jiang,^¶^ Peifen Lu,^¶^ Hui Li,^¶^ Xiaohong Song,^†^ and Jian Wu^¶^**

*†School of Physics and Optoelectronic Engineering, Hainan University, Haikou 570288,*

*China*

*‡School of Information and Communication Engineering, Hainan University, Haikou*

*570288, China*

*¶State Key Laboratory of Precision Spectroscopy, East China Normal University, Shanghai*

*200241, China*

**Center for Theoretical Physics, Hainan University, Haikou 570288, China*

***^§^****These authors contributed equally to this work.*

**^**^** *E-mail: wfyang@hainanu.edu.cn*

**S1. Adapted saddle-point equation**

Coulomb-corrected strong-field approximation (CCSFA) model fails in the case of electron wavepacket penetrating a finite potential barrier, where the electron suffers a strong binding effect from the Coulomb potential at a finite tunnelling exit, i.e., a dynamical potential energy. In other words, the effect of the Coulomb potential from the ionic cores has not been well included in the electron tunnelling process yet. Figure S1 shows the distribution of the initial tunnelling exit and the initial and final transverse momentum. The initial tunnelling exit of the electrons is symmetric along the major axis of the probe laser pulse, which is identical to an atomic ionization case. The neighboring Coulomb potential assisted tunnelling between the Ar and Kr is absent here (see Fig. S1(a)). The distribution of the initial transverse momentum is a Gaussian-like shape and the distribution of the final transverse momentum can trace the effect of the Coulomb focusing, both of which are similar with the case of atomic ionization (Fig. S1(b)).

However, the Improved-Coulomb-corrected strong-field approximation (ICCSFA) model provides access to all the initial velocity, tunnelling exit, ionization time of the electrons emerging at the continuum and the weight of the electron trajectories. The initial distribution of the birth time and transverse momentum along laser propagation direction and corresponding sub-barrier tunnelling dynamics along the image time are shown in Fig. S2. The direct electrons are ejected most probably at the negative peak of the electric field component along the major axis of elliptically polarized laser field (Fig. S2(a)). In comparison to the direct electrons, the capture electrons are emitted at the positive peak of the electric field component (Fig. S2(b)). Owing to the streaking of the elliptically polarized laser pulse, the direct and capture electrons at different moments of ionization will be ejected to different angles in the polarization plane of the laser field, which leads to different ratio of the direct and capture electrons at different angles. By measuring the width of the final momentum oriented perpendicular to the polarization plane of laser field, the ratio can be detected to reveal the physics of the resonant capture in molecular tunnelling (see Fig. 5 in the main text).

To gain insight into the underlying physics of the sub-barrier tunnelling dynamics, we show the photoelectron probability as a function of the image part of complex time in Figs. S2(c) and S2(d), respectively. As shown in Fig. S2(c), the maximum probability of the direct electron wave packet presents at *p*x_e_ = 0, which leads to the initial Gaussian-like distribution of transverse momenta at the tunnelling exit (see Fig. S2(e)). In contrast, the maximum probability of capture electrons occurs at *p*x_e_ ≈ ±0.5 a.u. (see Fig. S2(d)). As a result, the initial distribution of transverse momenta capture electrons presents two peaks at *p*x_e_ ≈ ±0.5 a.u. (Fig. S2(f)).

**S2. Initial tunnelling exit distributions**

Owning to the tracing ability of the initial tunnelling conditions of photoelectrons in the ICCSFA method, we can analyze the distribution of the electron tunnelling exit, electron trajectory, ionization probability and final momentum. Figure S3 shows the distributions of the tunnelling exit tagging to the interested photoelectron emission direction in the laser polarization plane of *pz*_e_ > 0 and *pz*_e_ < 0, where *y*_e_ is along the major axis of the elliptically polarized laser field. Most of photoelectrons in half plane of *pz*_e_ > 0 are born at the region *y*_e_<0 (see Fig. S3(a)), which means that the photoelectrons tunnels from Ar to Kr^+^. The electrons released between the cores of Ar and Kr^+^ will be resonantly captured by the Ar-Kr^+^. On the contrary, most photoelectrons with final momentum in half plane of *pz*_e_ < 0 are released from the region of *y*_e_>0 (see Fig. S3(b)), and these electrons are directly released to the continuum close to Ar site.

Figure S4 shows the FWHM width of *p*x_e_ as a function of the photoelectron emission angle *φ*_e_ in the area of *pz*_e_ < 0. The *p*x_e_ width distributions reconstructed by the widths of the capture and direct release electrons agree with the trajectory unresolved width distributions (see the red lines in Fig. 5(a) and (b) of the main text). The *p*x_e_^initial^ of electrons from capture region is much broader than that of direct released electrons (see Fig. 3(a) in the main text). The strong capture process will block the transfer of the initial momentum at tunnelling exit to the continuum, which leads to the narrowing of the photoelectron momentum distribution of strong capture electrons. Here, we define electrons that rotate around the nucleus more than five times as strong capture electrons. In contrast, weak capture electrons (rotation times ≤ 5) experience shorter duration of Coulomb focusing from the ionic Coulomb potential of Ar-Kr^+^ leading to a broader transverse momentum distribution than that of the strong capture process. Thereby, the FWHM width of *p*x_e_ is determined by the broadness of the initial momentum from capture region and the weak Coulomb focusing of the weak capture process. The direct released electrons present the narrowest width of final transverse momentum, while the width of strong capture electrons shows a relative broader distribution than that in direct release region. By utilizing ultrashort pulses, typically spanning only a few cycles or less, it becomes conceivable to selectively regulate the temporal access of fast weak capture electron species. In contrast to prolonged probe pulses, strong capture electrons remain bound for a duration surpassing that of the pulse itself, resulting in their effective suppression in the measured signal.

Figure S5 plots the ionization probability of the electron trajectory separated via its rotating times around the ionic cores of Ar-Kr^+^. In the photoelectron emission angle window of *φ*_e_ = (-180°, -150°), the weak capture, i.e., rotation times=2, dominate in the dynamics of photoemission, which leads to the maximum FWHM width of *p*x_e_ in the laser polarization plane (see Fig. S5(a) and Fig. 5 in the main text). As *φ*_e_ decreases from Figs. S5(a) to S5(c), the ratio of photoelectrons shows more rotation times resulting in the decreasing of the FWHM width of *p*x_e,_ which corresponds to a stronger and stronger Coulomb focusing effect. As shown in Figs. S5(d)-S5(f), the ratio of capture electrons decreases, and that of direct released electrons dramatically increase, which leads to the further decreasing of the width of *p*x_e_.

We performed an intensity scan from 50 TW cm-2 to 400 TW cm-2 corresponding to Keldysh parameter 1.6 (nonadiabatic tunneling) and 0.57 (adiabatic tunneling) with the ICCSFA simulations. Figure S6 shows the initial tunneling exit distribution of the second electron for cases with different laser intensities. The initial tunneling exit distributions of the strong/weak captured and direct released electrons are present for the cases with different laser intensities.

**S3. The temporal resolution of photoemission delays via angular streaking**

Considering the relationship between the transverse momentum distribution and the attosecond angular streaking, we then calculated the capture time of electrons with the statistically averaged treatment based on electron trajectories in ICCSFA simulation as a function of streaking angle *θ*, $\left\langle T_{\theta} \right\rangle=\frac{\sum_{i} \left[ \left( {tcon}_{i}-{t0}_{i} \right)\cdot W_{i} \right]}{\sum_{i} W_{i}}$. Here ${tcon}_{i}$, ${t0}_{i}$ and $W_{i}$ are the time in the continuum, the beginning time, and the weight of the *i*th trajectory in the region with streaking angle *θ* in the polarization plane. Figure S7 shows the reciprocal of the statistically averaged capture time $\frac{1}{\left\langle T_{\theta} \right\rangle}$as a function of photoelectron emission angle in the polarization plane, which presents the similar functional relationship with the transverse momentum width distribution ($\sigma_{px}^{e}$) in Fig. 5(b). To identify the electron capture process based on the protocol of the angular streaking, we employed the transverse momentum width angular distribution, instead of momentum distribution in the polarization plane, Figure S8 shows a typical three-dimensional trajectory of the captured electron. The electron is trapped in the potential well and moves around the ion. After some time, the electron breaks away from the core with the combined Coulomb and laser fields.


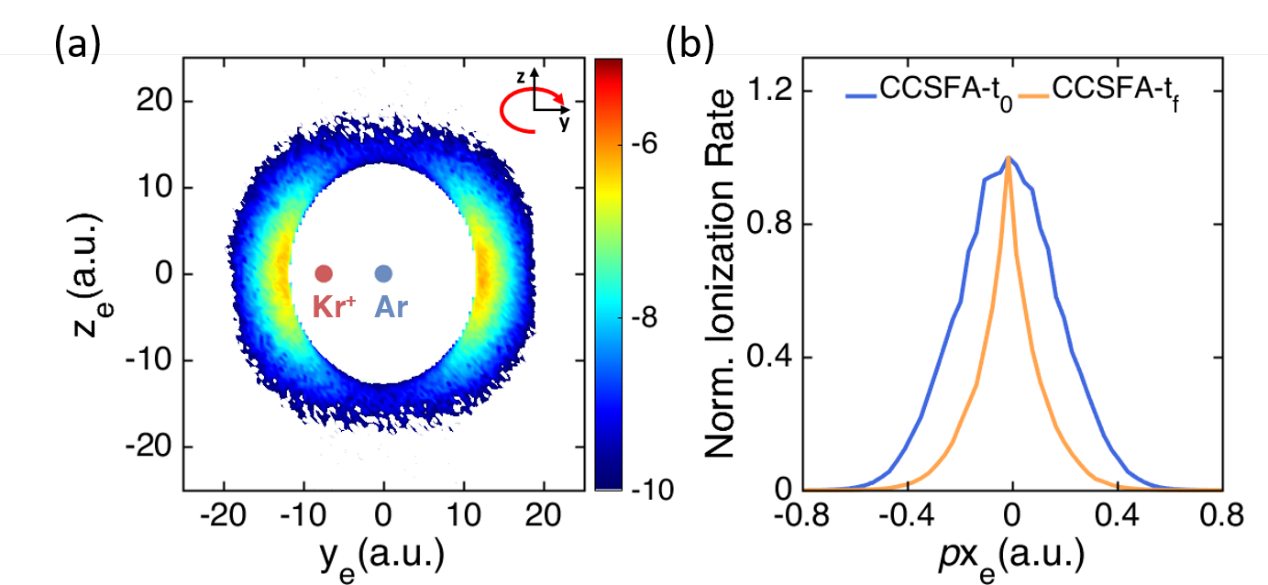


FIG. S1. Electron tunnelling condition simulated by CCSFA. (a) The distribution of electron tunnelling exit calculated via the CCSFA model released by a clockwise elliptically polarized laser field as the inset shows. (b) Normalized initial transverse momentum distribution (blue line) and final transverse momentum distribution (orange line) in the CCSFA calculation.


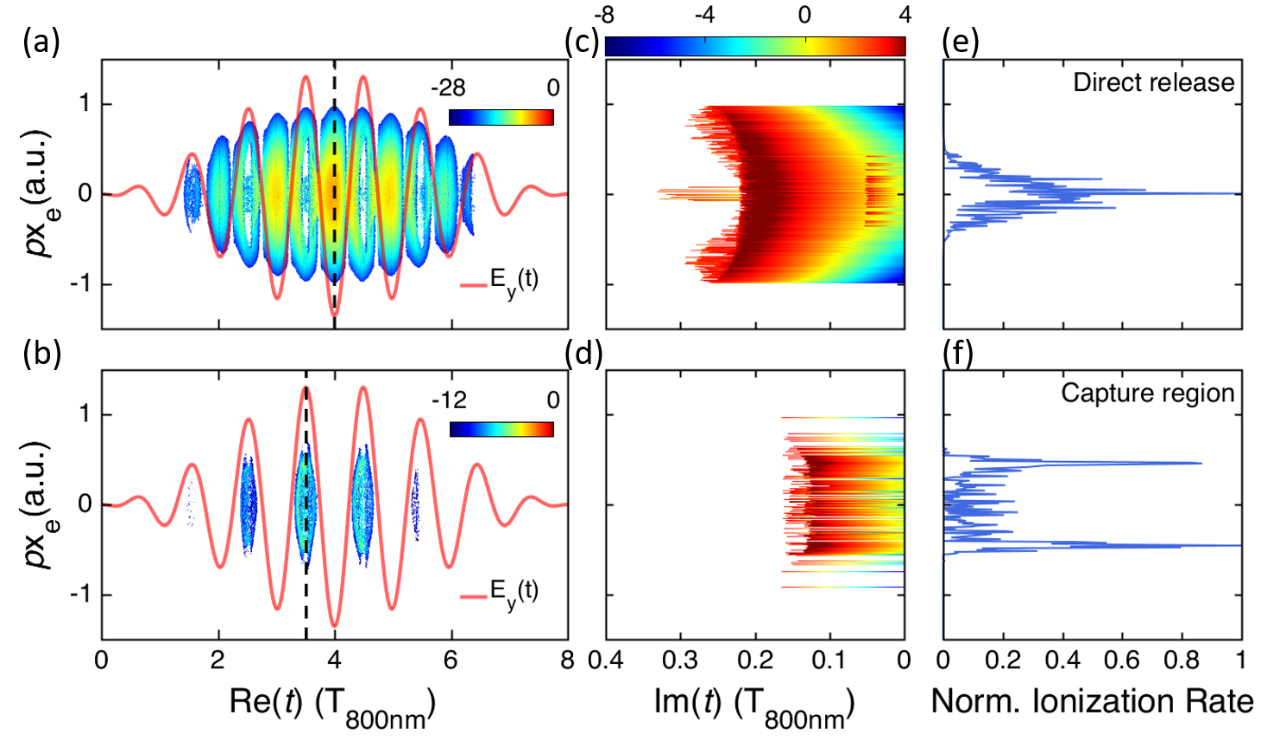


FIG. S2.  Tunnelling dynamics of photoelectrons from the direct and capture regions. (a), (b), Simulated birth time tagged ionization rate and transverse momentum along laser propagation direction from the direct release region and tunnelling capture region, respectively. The solid red curves denote the electric fields components along the major axis of elliptically polarized laser field. (c), (d), The electron wave packets propagate on the imaginary time axis, and the real times of the electron wave packets are denoted by the black dashed lines of a and b, respectively. (e), (f), The corresponding initial transverse momentum distribution at tunnelling exit with respect to (c), (d), respectively.


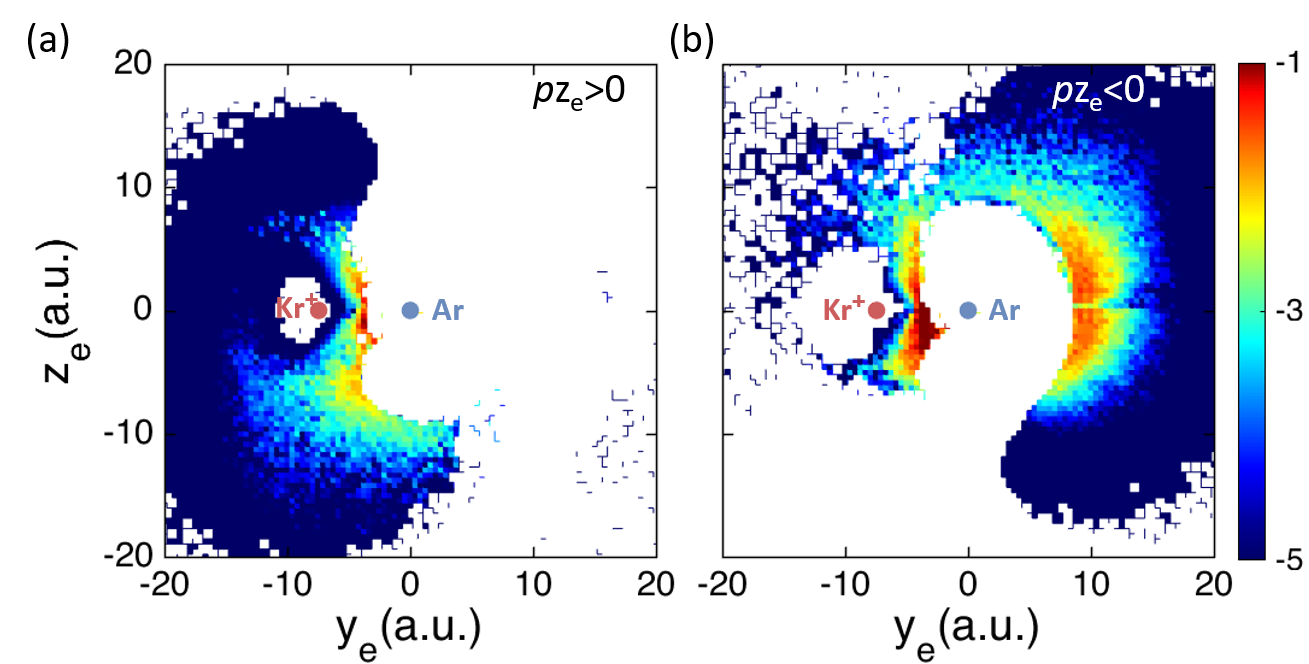


FIG. S3. Angular resolved initial tunnelling exit distribution for *pz*_e_ > 0 (a) and *pz*_e_ < 0 (b), respectively.


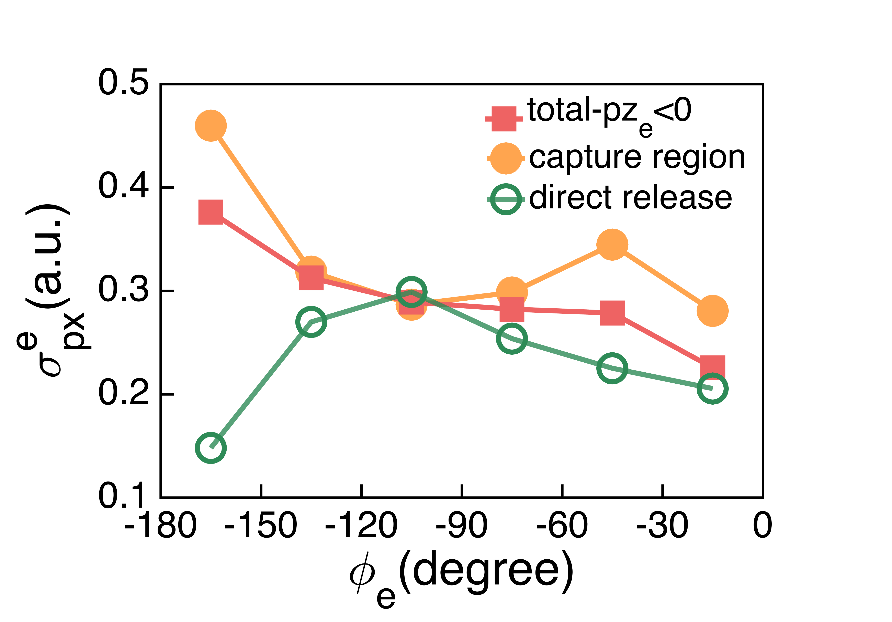


FIG. S4.  The trajectory resolved *px_e_* width distribution for *pz*_e_ < 0. The tunnelling capture and the direct release electrons are shown in solid orange circles and open green circles, respectively. The *p*x_e_ width distributions reconstructed by the widths of the capture and direct release electrons are shown in red squares.


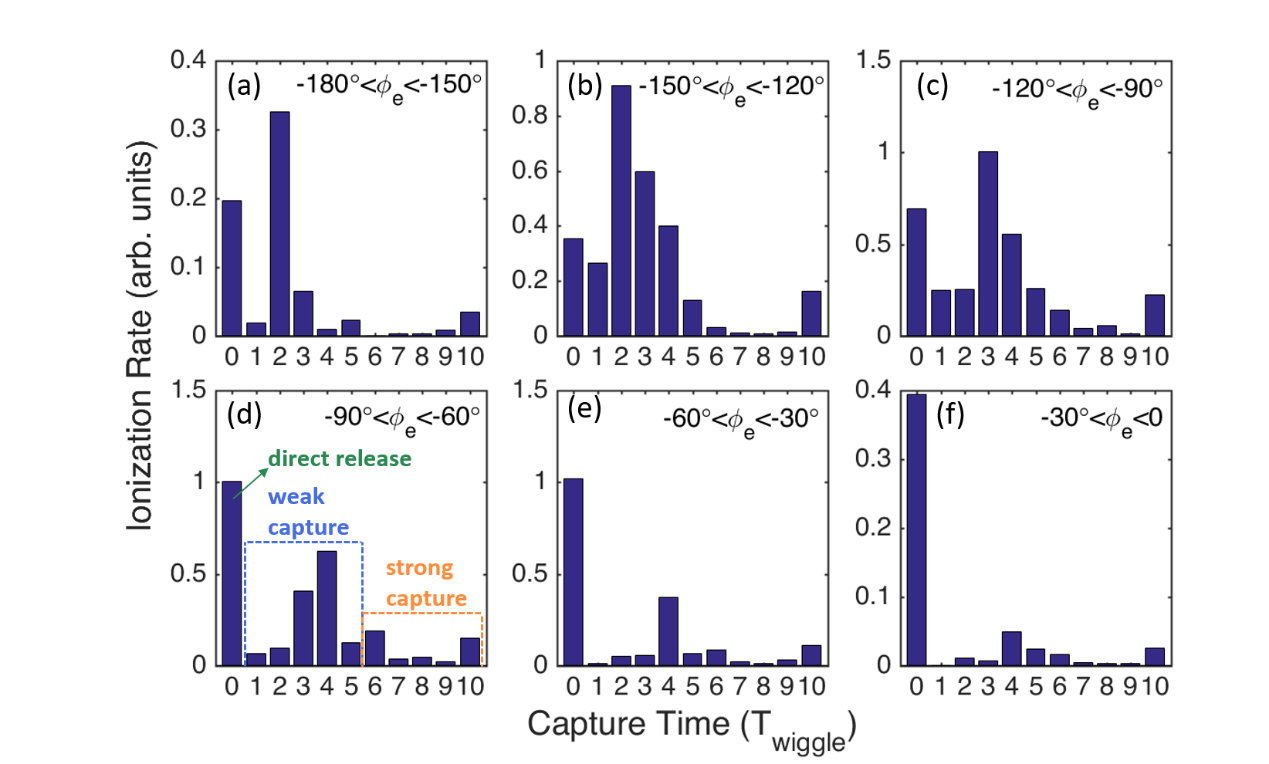
FIG. S5. Angular resolved electron trajectory distributions for *pz*_e_ < 0. The electron trajectory ionization rate distribution versus its rotation times around Kr^+^ (which is denoted as the capture times.) which is tagged to the photoelectron emission direction of *φ*_e_ = (a) -180°-150°, (b) -150°-120°, (c) -120°-90°, (d) -90°-60°, (e) -60°-30°, and (f) -30°-0 in the laser polarization plane, respectively.


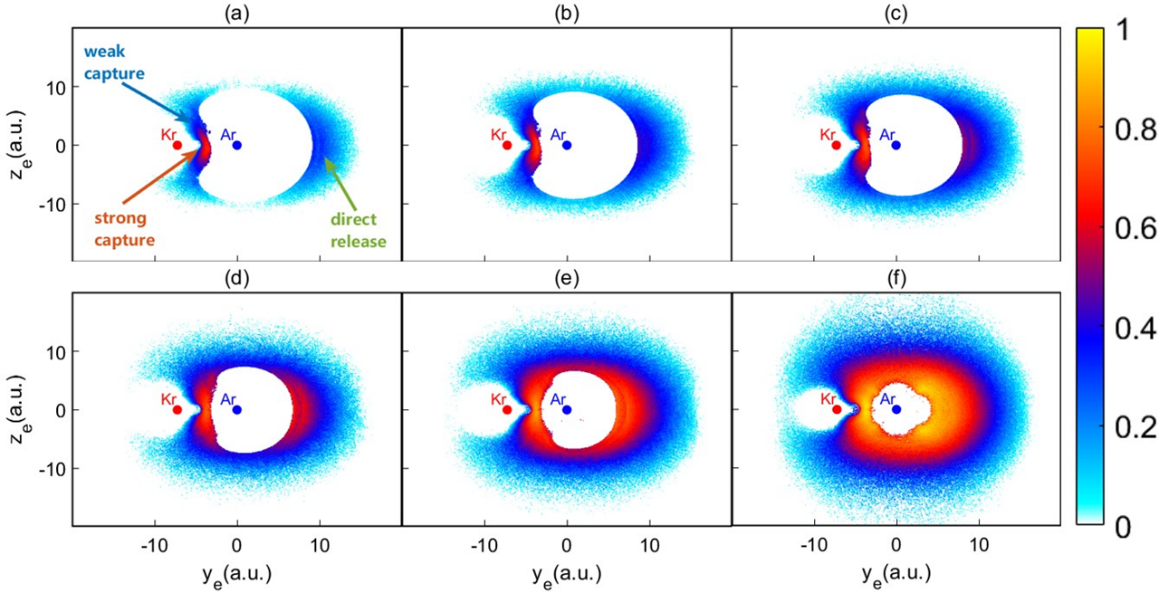


FIG. S6. The ICCSFA simulated initial tunneling exit distribution of the photoelectrons for the intensity scan from the peak intensity of (a) 50 TW cm-2, (b) 60 TW cm-2, (c) 70 TW cm-2, (d) 100 TW cm-2, (e) 120 TW cm-2, and (f) 400 TW cm-2.


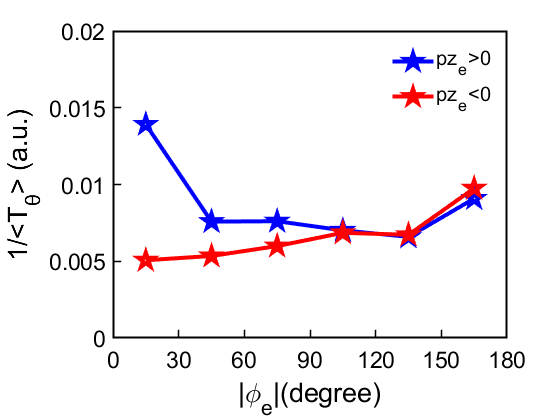


FIG. S7. The reciprocal of the statistically averaged capture time 1/〈*Tθ* 〉 is plotted as a function of photoelectron emission angle in the polarization plane. In this plot, points with *pz*_e_>0 are represented by blue pentagrams connected by lines, while points with *pz*_e_<0 are indicated by red pentagrams.


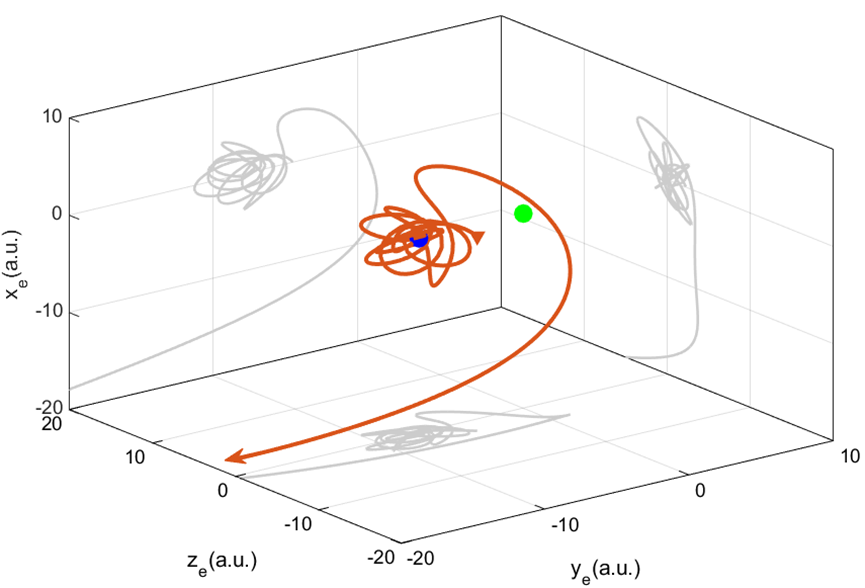


FIG. S8. A typical trajectory of the captured electron in three dimensions (the ye-ze plane is the polarization plane). The blue and green dots denote the positions of Kr+ and Ar+ ions, respectively. The orange triangle and arrow denote the start point and the direction of the electron trajectory.
